# Structure Analysis of Aerobic Granule from a Sequencing Batch Reactor for Organic Matter and Ammonia Nitrogen Removal

**DOI:** 10.3390/ijerph110302427

**Published:** 2014-02-26

**Authors:** Jun Li, Ang Cai, Danjun Wang, Chao Chen, Yongjiong Ni

**Affiliations:** College of Civil Engineering and Architecture, Zhejiang University of Technology, Hangzhou 310014, China; E-Mails: caiang123@163.com (A.C.); wangdanjun0509@163.com (D.W.); cc622368@126.com (C.C.); niyongjiong1984@163.com (Y.N.)

**Keywords:** aerobic granules, structure, freezing microtome, dissolved oxygen, microelectrode, CLSM

## Abstract

Aerobic granules were cultivated in a sequencing batch reactor (SBR). COD and ammonia nitrogen removal rate were 94% and 99%, respectively. The diameter, settling velocity and SVI_10_ of granules ranged from 2 to 5 mm, 80 to 110 m/h and about 40 mL/g, respectively. Freezing microtome images, DO concentration profiles by microelectrode, distribution of bacteria and EPS by confocal laser scanning microscopy (CLSM) show that the aerobic granules have a three-layer structure. Each layer has different thickness, character, bacteria, and DO transfer rate. A hypothesis for granule structure is proposed: the first layer, the surface of the granule, is composed mostly of heterotrophic organisms for organic matter removal, with a thickness range from 150 to 350 μm; the second layer, mostly composed of autotrophic organisms for ammonia nitrogen removal, with a thickness range from 250 to 450 μm; the third layer, located in the core of the granule, has mostly an inorganic composition and contains pores and channels.

## 1. Introduction

Aerobic granulation is an attractive biotechnology for wastewater treatment and has been commonly reported since the late 1990s [1,2]. Researchers intended to demonstrate that granular sludge has high settling velocities leading to good solid-liquid separation, high biomass retention, high activity, and an ability to withstand high loading rates [3,4,5,6].

Generally people think of granules as a special kind of biofilm. Aerobic biofilms were found to have a highly heterogeneous structure consisting of cell clusters and voids. Bacteria were primarily located in the cell clusters, embedded an extracellular polymeric substance (EPS) matrix. The voids form a network of pore channels connected with the bulk liquid [7]. The diffusion of substrates and oxygen forms a special structure with heterogeneous layers in the biofilm. A similar viewpoint of the structure showed aerobic granules had an aerobic zone and an anaerobic zone. Simultaneous nitrification and denitrification occurred in the aerobic granules [8,9]. Organic carbon was oxidized by heterotrophic organisms in the outer layer of the biofilm, whilst autotrophic organisms oxidized ammonia in the center layer in which oxygen was available but carbon substrate was absent [10,11].

High airflow rate and feast-famine regime for aerobic granulation causes the variation of substrates and oxygen in and out the biomass [12,13]. This could be important reason for the granule structure. However, a few experimental cases could provide evidence about the aerobic granule structure.

Oxygen microelectrode technology has received more attention for microorganism analysis. The tip of the microelectrode can be very small and has advantages of high resolution, high sensitivity, good selectivity and non-destructive and safe operation [14,15,16,17]. This work tried to analyze the structure of aerobic granules based on three methods that include freezing microtome sections, dissolved oxygen (DO) microelectrode and confocal laser scanning microscopy (CLSM).

## 2. Experimental Section

### 2.1. Experimental Setup

The aerobic sequencing batch reactor (SBR) was a cylinder with a working volume of 4.2 L and a diameter of 9 cm. The influent entered through the top of reactor, and a 2 L volume of the effluent was drawn. The reactor operated as follows: 30 min influence, 150 min aeration, 1 min settling, 6 min discharge and 24 min idling. The reactor operated for six cycles per day. The seed sludge was taken from the aeration tank in Garching Municipal Wastewater Treatment Plant in Munich, Germany. The reactors were fed with the acetate-based synthetic wastewater [18].

The volumetric organic load in this SBR was 4.2–5.5 kg COD/m^3^·d and the ammonium load was 225 g NH_4_-N/m^3^·d. The airflow rate was 300 L/h, which results in an upflow air velocity of 1.3 cm/s in the column. The temperature of operation was kept at about 17–22 °C.

### 2.2. Analytical Methods

Sludge volume index (SVI) and mixed liquid suspended solid (MLSS) were measured according to Standard Methods for the Examination of Water and Wastewater [19]. Chemical oxygen demand (COD_Cr_) and nitrogen were measured using Dr. Lange test kits following instruction of colorimetric standard method from Dr. Lange GmbH. Granules sample were taken from SBR from 178 to 225 day. Granules size and distribution of diameters were analyzed using a Leica digital optical microscope and Image-Pro Plus. Settling velocity of granules was measured in a cylinder (80 cm height) filled with tap water.

**Figure 1:**
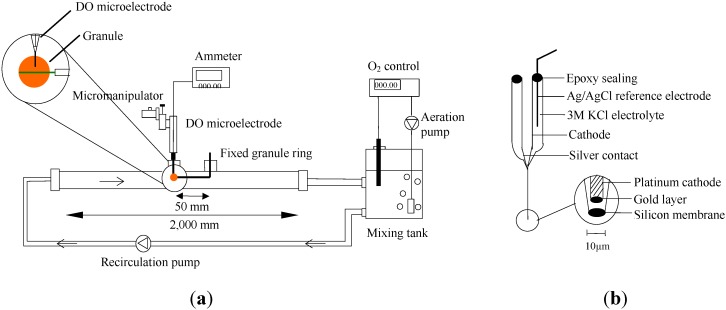
(**a**) Schematic diagram of the DO microelectrode measurement system; (**b**) DO microelectrode.

The measurement of oxygen concentration profiles in the granule was carried out outside the reactor in a special flow cell under defined hydrodynamic conditions using a microelectrode system (Figure 1). The flow cell was built similarly to a tube reactor used for biofilm cultivation [20,21]. The set-up consisted of a mixing tank with aeration unit and substrate solution, an aeration pump, a dissolved oxygen meter, a recirculation pump, an oxygen microelectrode, a micromanipulator, a tube reactor and an ammeter. The tank and pipes were made from Plexiglas. The flow velocity, substrate conditions and oxygen concentration could be controlled separately in the process of measurement. Diffusion of DO concentration under the steady state with 424 of Re numbers and different COD or NH_4_-N concentration were measured.

CLSM images had been recorded using a TCS SP confocal laser scanning microscope (Leica, Solms, Germany). Granule bacteria were stained using the SYTO 60 nucleic acid stain (Molecular Probes, Eugene, OR, USA). The stock solution of SYTO 60 as supplied was used at a dilution of 1:1,000 in de-ionised water. Glycoconjugates of biofilms were stained with the *Aleuria aurantia* lectin (Vector, Burlingame, CA, USA) conjugated to Alexa488 (Molecular Probes) according to a screening of all commercially available lectins on the same type of biofilms [22,23].

## 3. Results and Discussion

### 3.1. Formation and Characteristic of Granules

**Figure 2:**
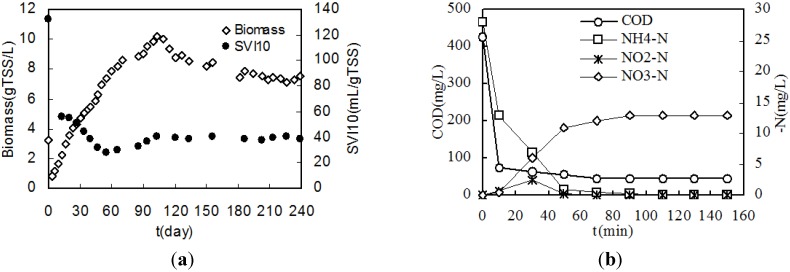
(**a**) Variation of the biomass concentration and SVI_10_; (**b**) Variation of COD, ammonia nitrogen, nitrite and nitrate in a typical SBR operation cycle.

Figure 2a shows the changes of biomass concentration and SVI with time. The first granule was found in the sixth day after inoculation. Later the biomass concentration in reactor increased constantly and reached maximum value of 11 g TSS/L till the 110th day and then decreases rapidly. After 130 days, the biomass concentration becomes stable at 7 g TSS/L. In the first 60 days, SVI decreased rapidly due to aerobic granulation, and it stabilizes at 40 mL/g TSS.

**Figure 3:**
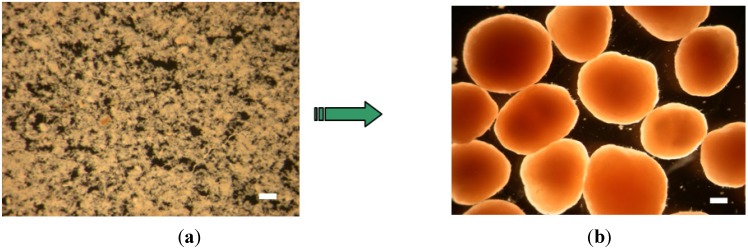
Microscopic examination of sludge and granules (scale bar = 1 mm). (**a**) Inoculum (floc sludge); (**b**) Aerobic granules at the 135th day.

Figure 2b shows the variation of COD, ammonia nitrogen, nitrite and nitrate in a typical SBR operation cycle. It indicates ammonia nitrogen deficit was after COD deficit. COD and ammonia removal rate were about 94% and 99%, respectively. Figure 3 shows two pictures of the inoculated activated sludge and aerobic granules by optic microscopy. It indicated that the granules had a smooth, dense and uniform surface structure.

### 3.2. Structure Measurement by DO Microelectrode

**Figure 4:**
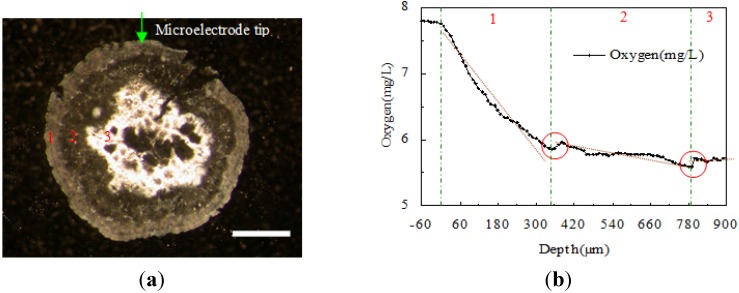
Granule section image by freezing microtome and DO profile by microelectrode system. (**a**) Image of a granule cross section (scale bar = 1 mm); (**b**) Profile of DO transfer variation in a granule in tap water.

Figure 4a shows a section picture of a granule which was frozen and then cut into sections in a freezing microtome. Three layers were observed in this granule. From the outside, the first layer had an even thickness and color, and it is relatively dense. The thickness of the second layer varied and it had a relatively loose structure. The third layer contained grey and white inorganic compounds and pores and channels. The pictures of different granules were analyzed with Image Pro software. The thickness of the first layer was about 150–350 μm, and that of the second one is about 250–450 μm.

Figure 4b shows the oxygen transfer and distribution curves when the granule was measured in tap water. From this picture, the oxygen concentration increased distinctly when the microelectrode went through the boundary between the first and second layer. A short time after the microelectrode went into the second layer, the oxygen concentration kept on decreasing. The same situation happened again when the microelectrode went through the boundary between the second and third layer. The DO decrease rate is mainly dependent on the DO consumption rate by bacteria and the diffusion resistance of the layers. Each layer has its own DO decrease rate. This indicates there are distinct different features in the three layers. DO profile by microelectrode shows a jump probably due to the fact the tip went through the boundary from the layer with higher DO decrease rate to the layer with a lower DO decrease rate.

Those two turning points (red rings) of the oxygen concentration curve indicate clearly the positions of the boundaries of those three layers. It implies the oxygen uptake rates of the three layers were different because of the variations in bacterias and components. The thickness of the first and the second layer were measured as 280 μm and 420 μm, respectively. This matches with the conclusion drawn from the same granule’s microscopy photo in Figure 4a. The granule section image shows a loose structure in the secondary layer and many pores in the third layer, respectively. Furthermore, Figure 4b shows the first layer has the highest DO decrease rate with depth partly due to higher diffusion resistance. These indicate the first layer is a more uniform and denser.

**Figure 5:**
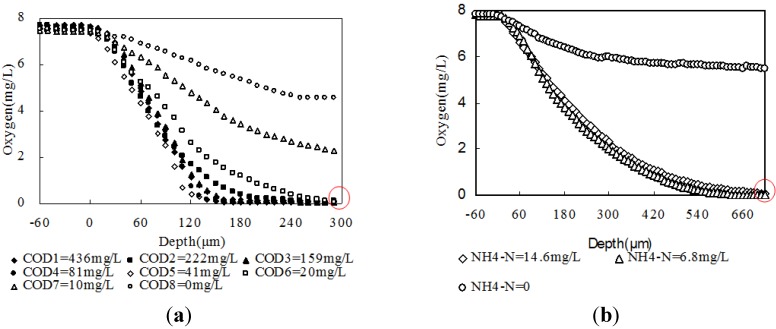
(**a**) DO concentrations variation in a granule in different COD; (**b**) DO concentrations variation in granule in different NH_4_-N.

Figure 5a shows DO concentration variations in the granule in different COD water mixed with only CH_3_COONa. DO was transferred deeper with the reduction of COD concentration and went through the whole granule under COD deficit. This point (red ring) occurred at about 310 μm depth and indicates that DO could be transferred to the deepest point when a carbon source existed. Figure 5b shows the DO concentration variation in a granule in different NH_4_-N water mixed with only NH_4_Cl. DO went through the whole granule under NH_4_-N deficit conditions. This point (red ring) occurred at about 750 μm depth and shows that DO could be transferred to the deepest point when NH_4_-N existed. This result matches the depth measurement by image observation. Furthermore, it suggests C degradation and deficit in the first layer and NH_4_-N nitrification and deficit in the second layer.

**Figure 6:**
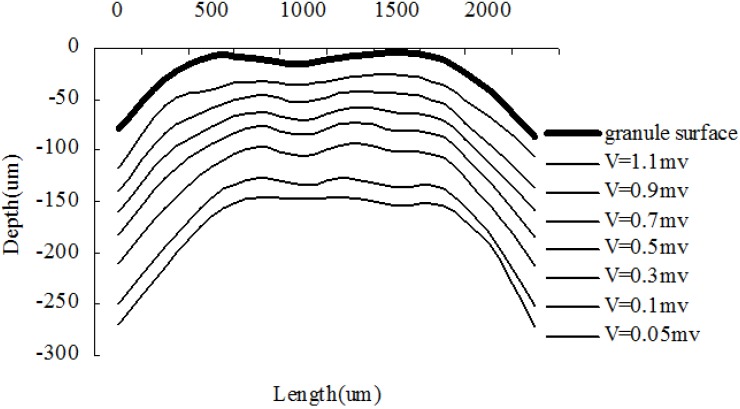
Equipotential line of DO microelectrode in the surface layer of a granule.

Figure 6 shows the equipotential lines of the DO microelectrode in the surface layer of a granule in water. The lines at different depth match with the profile of the granule surface. It indicates the first layer had a uniform structure.

### 3.3. Structure Measurement by CLSM

**Figure 7:**
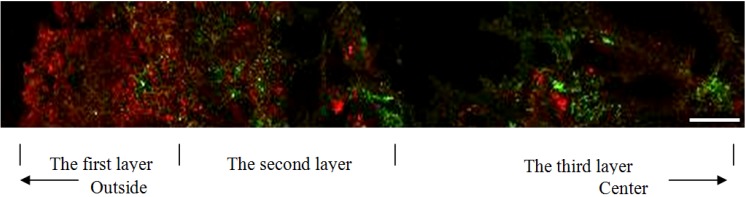
Distribution of bacteria (red) and EPS (green) in a granule by CLSM (scale bar = 0.1 mm).

CLSM was applied to analyze the distribution of bacteria and EPS in the granules. From Figure 7, it can be shown that the granules are composed of three layers. The first deep one is dense, composed mostly of bacteria. The second layer is relatively loose, but contains lots of both bacteria and EPS. The third layer is the loosest, filled with pores and composed of fewer bacteria and more EPS.

### 3.4. Analysis and Hypothesis of Granule Structure

All granules ranged in diameter from 2 to 5 mm and had a three-layer structure according to three analysis methods. The first layer around the surface and interior second layer were measured at a thickness ranging from 150 to 350 μm and 250 to 450 μm, respectively. The third layer from the boundary of the second layer to the center showed various areas depending on size of granules.

**Figure 8:**
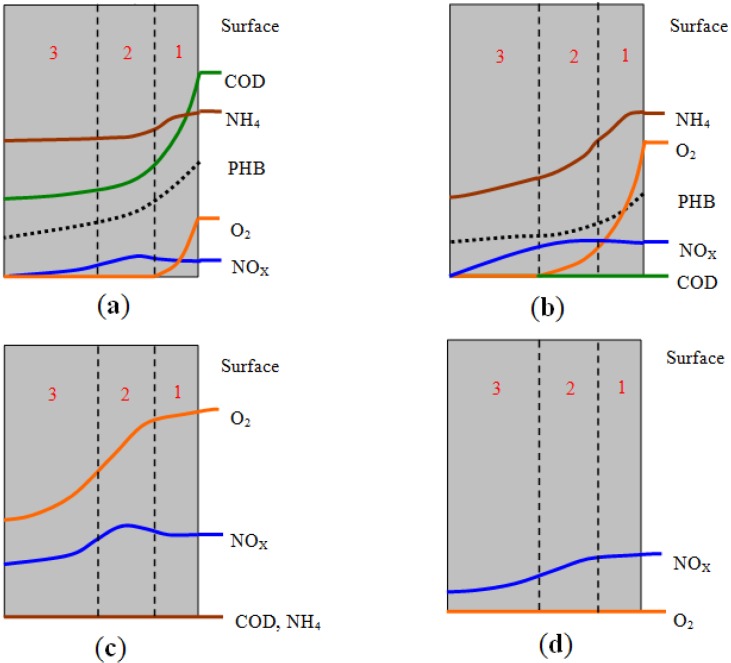
Schematic profiles of substances transfer inside the granule. (**a**) Beginning of aeration; (**b**) Carbon deficit; (**c**) Ammonia deficit; (**d**) Idle period. Curves represent variation of substances concentration in different depths.

At the beginning of aeration, the carbon concentration in the reactor is quite high, and the part of carbon could diffuse and go into the interior of granules and be synthesized as PHB by microorganisms and stored inside the cells. With the aeration process, COD concentration decreases quickly. At the same time, partial nitrification happens in the first aerobic layer. Before the COD concentration becomes zero, oxygen can only penetrate the first layer and then there is an anoxic zone in the second and third layers. Part of the nitrite or nitrate is denitrified in the second and third layers utilizing PHB as shown in Figure 8a. It indicates that surface of the granule is mostly composed of heterotrophic organism, because autotrophic bacteria are disadvantaged at high carbon concentration.

With COD concentration decrease and deficit, the speed of nitrification increases and a great deal of ammonium is eliminated. Oxygen penetrates till the second layer, shown in Figure 8b. Finally ammonium concentration decreases to zero. It indicates autotrophic organisms could come into being dominantly in the second layer as carbon concentration decreases.

Figure 8c shows oxygen can penetrate the third layer into the core of the granule when ammonium reaches a deficit. In this period, the microorganisms undergo endogenetic respiration. This indicates there are few organisms in the core because of there are no substances and the long term endogenetic respiration. Because there is a relatively long idle period during the operation in this SBR, the internal carbon source in granules could be utilized to eliminate a certain amount of nitrate by denitrification, shown in Figure 8d. This occurred similarly in the influence period. The above analysis and hypothesis can also illustrate the three layers structure and mechanism of organic matter and ammonia biodegradation in the aerobic granule.

## 4. Conclusions

Aerobic granules were successfully formed in an SBR for organic matter and ammonia nitrogen removal. The granule structure could be measured and analyzed by freezing microtome, DO microelectrode and confocal laser scanning microscopy (CLSM). The DO microelectrode is an efficient and sensitive system to prove the structure and biodegradation of substances in the granule. CLSM images show the distribution of bacteria and EPS to distinguish the differences between the three layers. These methods can measure the thickness and characters of layers in the granule. Three layers are formed because of mass change and transfer in the granules and the competition of microorganisms.
